# Supplementary material for: Brimonidine Eye Drops within the Reach of Children: A Possible Foe
Source: Children (Basel). 2024 Mar 7;11(3):317. doi: 10.3390/children11030317 (PMC10969194; doi:10.3390/children11030317)
Supplement: Supplementary file 1 [file children-11-00317-s001.zip › children-2871963-supplementary.pdf]

children

MDPI

Supplementary Material of:

Brimonidine Eye Drops within the Reach of Children: A Possible Foe

Daniela Trotta [1,†], Mirco Zucchelli [2,3,†], Carmela Salladini [1], Patrizia Ballerini [2,3], Claudia Rossi [2,3] and Maurizio Aricò [1,*]

[1] Department of Pediatrics, Santo Spirito Hospital, Azienda Sanitaria Pescara, 65121 Pescara, Italy; daniela.trotta@asl.pe.it (D.T.); carmela.salladini@asl.pe.it (C.S.)
[2] Center for Advanced Studies and Technology (CAST), "Gabriele d'Annunzio" University of Chieti-Pescara, 66100 Chieti, Italy; m.zucchelli@unich.it (M.Z.); patrizia.ballerini@unich.it (P.B.); claudia.rossi@unich.it (C.R.)
[3] Department of Innovative Technologies in Medicine and Dentistry, "Gabriele d'Annunzio" University of Chieti-Pescara, 66100 Chieti, Italy
* Correspondence: maurizio.arico@asl.pe.it
† These authors equally contributed to the work.

Brimonidine quantification in urine and plasma sample by UPLC-MS/MS analysis

Materials and sample preparation

Brimonidine and internal standard Brimonidine-d4 L-Tartrate (Toronto Research Chemicals) were purchased from Spectra2000 Srl. Brimonidine powder was dissolved in methanol and internal standard Brimonidine-d4 L-Tartrate in water. Internal standard working solutions (100 ng/mL and 2 ng/mL for urine and plasma analysis, respectively) were freshly prepared by diluting the IS stock solution with acetonitrile. Calibrators and Quality Controls (QCs) were prepared in pooled human plasma (anticoagulant K3EDTA) and pooled human urine by standard addition of Brimonidine.

Plasma calibrators were prepared over the range 0-10 ng/mL, with QCs at 0.75, 3 and 7.5 ng/mL for low, mid, and high levels, respectively.

Urine calibrators were prepared over the range 0-500 ng/mL, with QCs at 3, 75 and 375 ng/mL for low, mid, and high levels, respectively.

UPLC- MS/MS analysis for brimonidine quantification in urine and plasma

Table S1. LC gradient.

Time (min)	Flow rate (mL/min)	%A	%B	Curve
**0**	0.5	95	5	initial
**1.00**	0.5	95	5	6
**2.00**	0.5	5	95	6
**3.00**	0.5	5	95	6
**3.01**	0.5	95	5	6
**6.00**	0.5	95	5	6

Table S2. MRM parameters for Brimonidine and internal standard Brimonidine-d4.

Compounds	Transitions (*m*/*z*)	Dwell (secs)	Cone (V)	Collision energy (eV)
**Brimonidine (quantifier)**	292.2>212.2	0.100	50	27
**Brimonidine (qualifier)**	292.2>249.2	0.100	50	27
**Brimonidine-d4 (IS)**	296.2>216.2	0.100	50	27

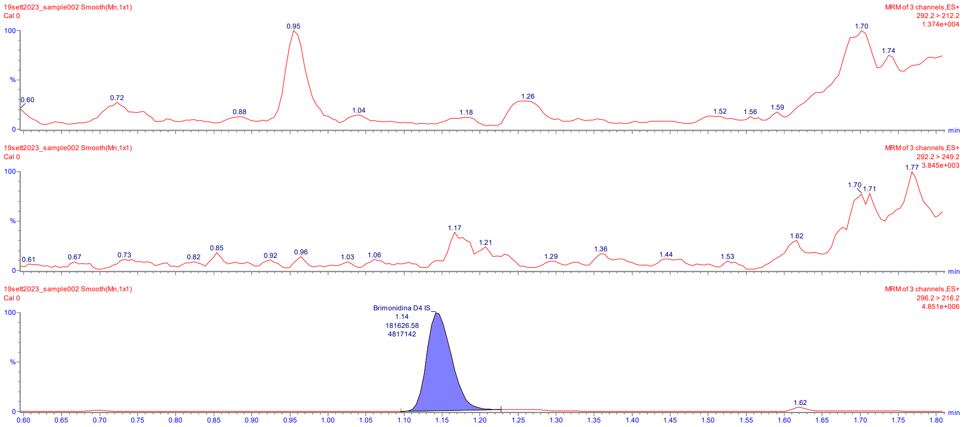

Figure S1. Chromatographic separation of brimonidine in a urine sample used as a blank sample type. Chromatographic separation of brimonidine (quantifier), brimodine (qualifier), and internal standard Brimonidine-d4 in a urine sample used as a blank urine in the optimization step.

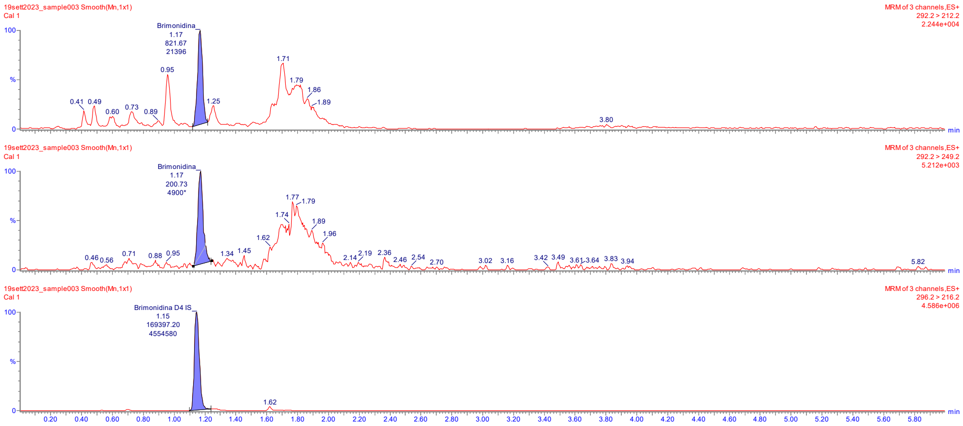

Figure S2. Chromatographic separation of brimonidine in a blank urine spiked sample. Chromatographic separation of brimonidine (quantifier), brimodine (qualifier), and internal standard Brimonidine-d4 in a urine blank sample spiked with Brimonidine 1 ng/mL.

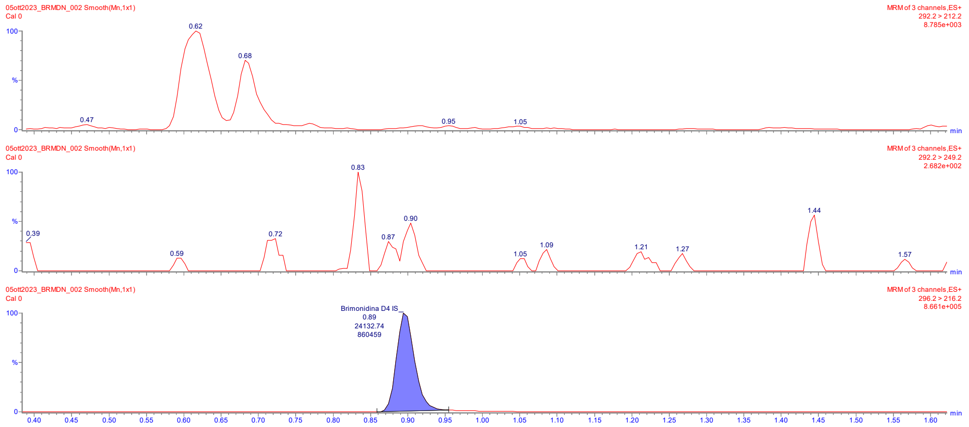

Figure S3. Chromatographic separation of brimonidine in a plasma sample used as a blank sample type. Chromatographic separation of brimonidine (quantifier), brimodine (qualifier), and internal standard Brimonidine-d4 in a plasma sample used as a blank plasma in the optimization step.

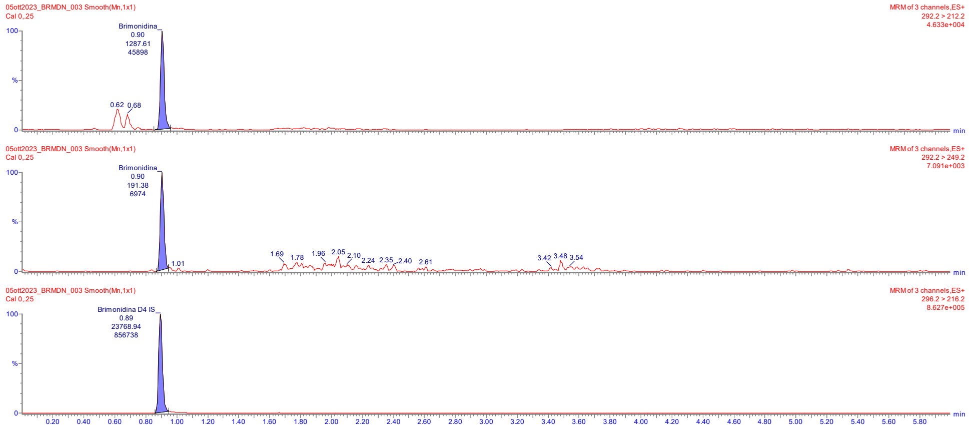

Figure S4. Chromatographic separation of brimonidine in a blank plasma spiked sample. Chromatographic separation of brimonidine (quantifier), brimodine (qualifier), and internal standard Brimonidine-d4 in a plasma blank sample spiked with Brimonidine 0.25 ng/mL.
